# Effects of a multicomponent high intensity exercise program on physical function and health-related quality of life in older adults with or at risk of mobility disability after discharge from hospital: a randomised controlled trial

**DOI:** 10.1186/s12877-020-01829-9

**Published:** 2020-11-11

**Authors:** Sylvia Sunde, Karin Hesseberg, Dawn A. Skelton, Anette Hylen Ranhoff, Are Hugo Pripp, Marit Aarønæs, Therese Brovold

**Affiliations:** 1 Institute of Physiotherapy, OsloMet - Oslo Metropolitan University (OsloMet), PO Box 4, St. Olavs Plass, 0130 Oslo, Norway; 2 Diakonhjemmet Hospital, PO Box 23, Vinderen, 0319 Oslo, Norway; 3 School of Health and Life Sciences, Glasgow Caledonian University, Glasgow, UK; 4 Department of Clinical Science, University of Bergen, Bergen, Norway; 5 Faculty of Health Sciences, OsloMet - Oslo Metropolitan University (OsloMet), Oslo, Norway

**Keywords:** Hospitalisation, Physical function, Health-related quality of life, Older adults, Exercise interventions

## Abstract

**Background:**

Many older people suffer from mobility limitations and reduced health-related quality of life (HRQOL) after discharge from hospital. A consensus regarding the most effective exercise-program to optimize physical function and HRQOL after discharge is lacking. This study investigates the effects of a group-based multicomponent high intensity exercise program on physical function and HRQOL in older adults with or at risk of mobility disability after discharge from hospital.

**Methods:**

This single blinded parallel group randomised controlled trial recruited eighty-nine home dwelling older people (65–89 years) while inpatient at medical wards at a general hospital in Oslo, Norway. Baseline testing was conducted median 49 (25 percentile, 75 percentile) (26, 116) days after discharge, before randomisation to an intervention group or a control group. The intervention group performed a group-based exercise program led by a physiotherapist twice a week for 4 months. Both groups were instructed in a home-based exercise program and were encouraged to exercise according to World Health Organisation’s recommendations for physical activity in older people. The primary outcome, physical performance, was measured by the Short Physical Performance Battery (SPPB). Secondary outcomes were 6-min walk test (6MWT), Berg Balance Scale (BBS), grip strength, Body Mass Index (BMI), and HRQOL (the Short-Form 36 Health Survey (SF-36)). Data were analysed according to the intention-to-treat principle. Between-group differences were assessed using independent samples t-test.

**Results:**

The groups were comparable at baseline. Intention-to-treat analysis showed that the intervention group improved their functional capacity (6MWT) and the physical component summary of SF-36 significantly compared to the control group. No further between group differences in change from baseline to 4 months follow-up were found.

**Conclusions:**

A high intensity multicomponent exercise program significantly improved functional capacity and physical HRQOL in older adults with or at risk of mobility disability after discharge from hospital. The study suggests that this population can benefit from systematic group exercise after hospital-initial rehabilitation has ended.

**Trial registration:**

ClinicalTrials.gov. NCT02905383. September 19, 2016.

**Supplementary information:**

**Supplementary information** accompanies this paper at 10.1186/s12877-020-01829-9.

## Background

Reduced physical function with increasing age can have tremendous negative consequences, both for the older individuals, their families and the society, hence preserving physical function, independence and HRQOL in older adults is central to the global response to population ageing [1]. Hospitalization often exacerbates the effects of ageing on physical function, and many older people suffer from mobility limitations and reduced HRQOL after discharge [2–5].

The literature on interventions aiming to preserve physical function [6–9] and HRQOL in the general population is vast, and interventions should include endurance, strength, balance and flexibility exercises [10]. To preserve good health, the World Health Organization (WHO) recommends people aged 65 years and older to engage in cardiorespiratory exercise training of moderate intensity at least 150 min per week, or at least 75 min if the intensity is vigorous [11]. A combination of moderate- and vigorous-intensity exercise is just as good, and bouts of aerobic exercise lasting at least 10 min count in the weekly summary. In addition, muscle-strengthening exercises, involving major muscle groups, should be conducted at least twice a week. Further, older adults with poor mobility are recommended to perform physical activity to enhance balance and prevent falls on at least 3 days per week. Those who are not able to meet the recommendations due to health conditions are encouraged to engage in as much physical activity as their abilities and conditions allow [11].

Physical activity and exercise therapy after hospitalization of geriatric patients has shown to be feasible [12] and could be an important means to help counteract the challenges associated with an ever-growing older population [13–17]. Nevertheless, a consensus regarding the most effective exercise-program to optimize physical function and HRQOL after discharge from hospital is lacking [18–20]. However, the rehabilitation sessions should be supervised to increase adherence [12] and reduce falls [21]. Furthermore, interventions with high intensity has proven somewhat superior to interventions with lower intensity in terms of improving physical function in community-dwelling older adults with impaired mobility, physical disability and/or multi-morbidity [22]. A recent systematic review on effectiveness of interventions to prevent pre-frailty and frailty progressions in older adults found that the group-setting was crucial to the effect of physical exercise programs [23].

By targeting older people with or at risk of mobility disability while inpatient, the vicious circle of inactivity and reduced physical function and HRQOL often experienced after discharge for older people could be counteracted [15]. Therefore, the objective of this study is to examine the effects of a high intensity multicomponent group-based intervention on physical function and HRQOL in older adults with or at risk of mobility disability after discharge from hospital.

## Methods

### Study design

A parallel group randomised controlled trial with one intervention group and one control group, allocated on a 1:1 ratio. The CONSORT 2010 Statement are followed in our report [24]. See Additional file 5 for CONSORT 2010 Checklist. The associations between the participants baseline scores on HRQOL (SF-36) and physical function (SPPB) is submitted as an independent article but it is still not accepted for publication.

### Setting and participants

Participants were initially recruited while acutely admitted to a general hospital in Oslo, Norway. Recruitment was based on registration lists of patients admitted at four medical wards. The recruitment period was from September 2016 to May 2019. Baseline testing was conducted after discharge and when the participants had completed hospital-initial rehabilitation. Participants provided written informed consent.

Inclusion criteria: age ≥ 65 years, live independently in the community, be at risk of mobility disability with a Short Physical Performance Battery (SPPB) score of < 10 while inpatient [25], ambulate independently (walking aid permitted), and understand Norwegian language. Further, they had to be assessed by a doctor (A.H.R or M.A.) as eligible for the intervention according to the standards from the American Heart Association [26].

Exclusion criteria: moderate or severe cognitive disorder (Score on Mini Mental State Examination < 20) [27], life expectancy less than 8 months, exercise regularly more than twice a week at a fitness centre or in a structured exercise program.

All participants received routine care, discharge planning, follow-up care and rehabilitation normally provided.

Changes to methods after trial commencement: we started the study with age ≥ 70 as an inclusion criterion but altered it to ≥65 after 1 year as an attempt to increase the recruitment speed. Further, the intervention was planned to be performed in cooperation with physiotherapists working in primary health care and in localities offered by three different city districts in Oslo Municipality, to be close to the participants homes and facilitate implementation of the intervention after the study had ceased. However, due to poor recruitment rate that made it counterproductive to run the intervention at three different sites, we decided to merge the groups and offer the intervention at the hospital gym.

### Intervention

The intervention group performed a group-based high-intensity multicomponent exercise program twice a week for four to 5 months, maximum 32 sessions or 5 months. The intervention was based on the Norwegian Ullevaal model [28] and the Swedish High-Intensity Functional Exercise Program (the HIFE program) [29, 30]. The intervention was led by one or two physiotherapists, in groups of 2–10 participants. The participants performed two strength exercises for the lower limbs (standing-up from sitting in a parallel stance and forward lunges), six balance exercises (walking forward on a line on a flat surface, heel raises, reaching for an object in various directions, one leg standing, step-over, and throwing and catching a ball), in addition to trunk rotation while seated. The exercise program is described in detail in Additional file 1, in accordance with the CERT-recommendations [31]. The exercises were accompanied by music, and conducted in the same sequence each session, following a detailed manual designed in accordance to a 53-min long playlist.

The intensity of the exercise was self-paced, but the participants were encouraged to exercise progressively, with a gradual approach to high intensity. High-intensity strength exercises were defined as two sets of 8–12 repetition maximum (RM) and the balance exercises were performed near the limits of maintaining postural stability [30]. The exercises were adjusted according to the participants’ health status, in each session. The participants wore weighted belts around the waist for the two strengthening exercises, loaded with a maximum of 12 kg. Each session also contained three 6–9 min bouts of high intensity endurance training, where the participants were encouraged to exercise with a Borgs exertion of 15–18 [32] the last 3–4 min, interspersed with flexibility exercises, in addition to the strength and balance exercises. Adverse events were registered in the following four categories; falls, cardiovascular events, musculoskeletal injuries and health care utilization [33].

Both the intervention group and the control group were given written information in Norwegian on the recommendations from the WHO on physical activity for people aged 65 and above [11], and they were encouraged to adhere to this recommendations (see Additional file 2). Both groups were instructed in a home-based exercise program to improve strength of the lower extremities and balance (see Additional file 3). This exercise program was developed by physiotherapists from the Norwegian University of Science and Technology (NTNU) and Trondheim Municipality in 2016, founded by the Norwegian Directorate of Health [34]. The participants were encouraged to perform the exercises at least 3 days a week. These exercises were also included in the multicomponent high intensity exercise intervention, so the intervention group was encouraged to perform the exercises at least once a week at home if they had attended the intervention group twice that week.

### Outcome measures

Information about the participants’ age, sex, living status (alone or with someone), education, hospital discharge diagnoses, number of comorbidities at the time of discharge, and length of stay were recorded from the participants` hospital records and by asking the participants. In addition, the participants filled out the International Physical Activity Questionnaire – Short form (IPAQ-SF) at home before baseline testing [35]. Assessments were conducted at a hospital outpatient clinic, by trained research assistants blinded to the group allocation, at baseline and following the intervention at 4 months.

#### Primary outcome measure

Physical performance was measured using the Short Physical Performance Battery (SPPB), a performance based test that evaluates balance (ability to stand with feet together in side-by side, semi tandem and tandem positions), functional mobility (gait speed; time to walk 4 m in preferred tempo) and muscle strength (time to rise from a chair and return to the seated position five times) [36]. The sum score ranges from 0 to 12 (worse-best).

#### Secondary outcome measures

Functional capacity was measured by the six-minute walk test [37], performance-based balance by Berg Balance Scale (BBS) [38], muscle strength (grip strength) by Jamar dynamometer [39], weight and Body Mass Index (BMI) by a Tanita BC-418 Body Composition Analyzer for the participants without a pacemaker (contraindication). An electronic body scale was used for patients with a pacemaker, and BMI was calculated (weight in kilograms divided by height in meters squared). Finally, HRQOL was measured by the Medical Outcome Study 36 Item Short-Form Health Survey, version 2 (SF-36) [40].

#### Sample size

A medium meaningful difference between the groups in change of SPPB was defined to 0.75 points with an expected standard deviation of 1.48 points [41]. To obtain 80% statistical power with a 5% significance level, 126 participants, 63 in each group, was needed. We aimed to include at least 150 participants, to compensate for potential drop-outs.

### Randomisation

Allocation to an intervention group or a control group was done after baseline testing, based on a computer-generated permuted block randomization scheme. Each block contained between four to ten subjects. TB and KH administered the scheme, and sealed envelopes were used.

### Statistical analyses

Statistical analyses were conducted with the IBM SPSS version 25 (SPSS Inc., Chicago, IL). *P*-values < 0.05 were considered statistically significant and all tests were two-sided. The normality of the distributions was examined graphically by histograms and Q-Q plots, and by comparing the mean with the median. Data are described as means and standard deviations (SD) for normally distributed continuous variables, and median and quartiles (25, 75) for a continuous variable that was skewed (length of stay). Categorical variables are described with proportions and percentages.

Between group differences in change from baseline to follow-up were analysed using the independent samples t-test according to the intention to-treat (ITT) principle. The ITT analyses were also conducted on a dataset where missing values were substituted by using the multiple imputations function in SPSS [42]. Floor and ceiling effects were considered when more than 20% of the participants achieved the lowest or highest score. Effect size was calculated, and interpreted according to the guidelines proposed by Cohen [43]; .2 = small effect, .5 = medium effect and .8 = large effect.

### Research ethics

The Regional Ethics Committee for Medical Research approved the study (REK 2015/2432), and the trial was registered at ClinicalTrials.gov in September 2016, NCT02905383. The first patient was included in September 2016.

## Results

### Flow of participants

**Fig. 1:**
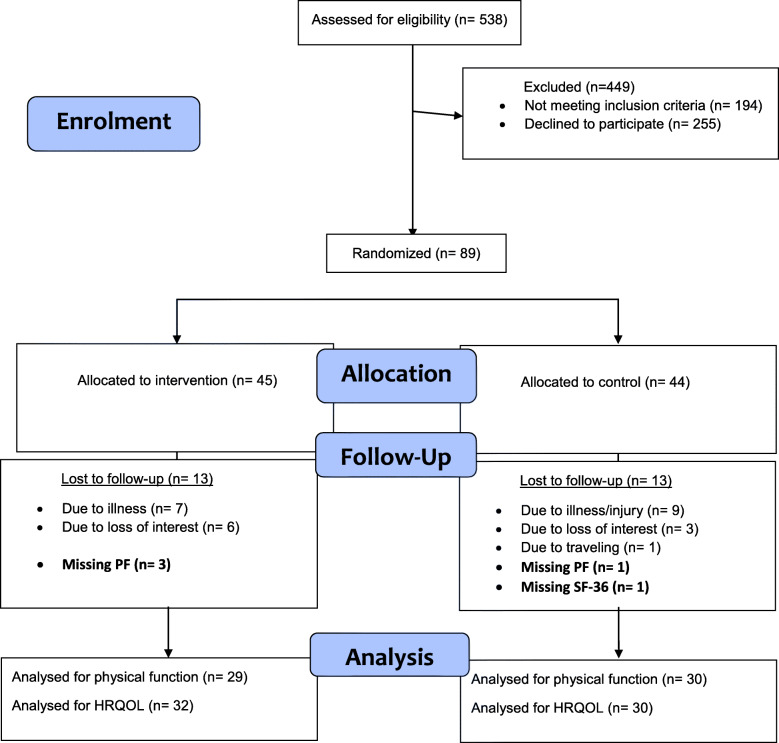
Flow diagram through 4 months follow-up. Missing PF means that the participant did not perform the tests for physical function but filled out the self-reported questionnaire (SF-36). Missing SF-36 means that the participant performed the tests for physical function but did not fill out SF-36

The flow of the study participants can be seen in a flow diagram (Fig. 1). Five hundred and thirty-eight participants were screened for eligibility, of which 89 were included. One hundred and ninety-four did not meet the inclusion criteria, and 255 refused to participate. The most common reasons for declining to participate were regular physical training at fitness centre, too busy helping kin or others, did not want regular appointments twice a week, traveling time/logistics and spending time abroad. Recruitment stopped before the sample size target was reached due to a slow recruitment rate and a limited timeframe. Baseline testing was conducted median 49 (25 percentile, 75 percentile) (26, 116) days after discharge. Forty-five participants were randomly allocated to the intervention group and 44 to the control group. The groups were comparable at baseline. One man in the intervention group withdrew from the study and requested that we deleted his data. Additional twelve participants (28.9% in total) in the intervention group and thirteen participants (29.6%) in the control group were lost to follow-up.

Twenty-six (89.7%) of the 29 participants with data from the four-month follow-up attended at least 16 (50%) of the group-based sessions and were defined as adherent to the intervention. These 26 participants attended mean (SD) 28.1 (3.9) sessions, while the mean (SD) for the total 29 participants was 25.5 (8.6) sessions. Reasons for not attending the sessions were experiencing decline in health or re-hospitalisation. The Borg scale and repetition maximum (RM) were used to encourage the participants to achieve high intensity during the endurance and strength exercises respectively. For the balance exercises the base of support was reduced and the ball was thrown in a more challenging manner. However, adherence regarding actual intensity accomplished was not measured.

Adverse events during exercise: the physiotherapists leading the intervention group reported three falls without injury. One participant was confused (delirium) at a session and was admitted to the hospital.

#### Participant characteristics

Table 1 shows the baseline characteristics for the total sample and for the intervention group and the control group separately. There were no statistically significant differences in the participants who were included in the study and those who were excluded regarding sex and age. The participants who were lost to follow-up scored significantly worse on SPPB (*p* = 0.026) and six min walk test (*p* = 0.033) at baseline than the rest of the sample. No other significant differences were found in characteristics or outcome measures between completers/dropouts at baseline.

**Table 1:** Characteristics of the study sample. Means, standard deviations (SD), numbers and percentages

Characteristics	Total (*N* = 88)	Intervention group (*n* = 44)	Control group (*n =* 44)
Age in years, mean (SD)	78.3 (5.5)	78.6 (5.7)	77.9 (5.2)
Sex, female n (%)	43 (48.9)	17 (38.6)	26 (59.1)
Living alone, n (%)	45 (51.1)	22 (50.0)	23 (52.3)
Education, n (%)			
Less than bachelor’s degree	35 (39.8)	12 (27.3)	23 (52.3)
Bachelor’s degree	32 (36.4)	20 (45.5)	12 (27.3)
More than bachelor’s degree	21 (23.9)	12 (27.3)	9 (20.5)
Length of stay, in days, median (IQR)	2 (1–4)	2 (1–4.8)	3 (1–4)
Number of comorbidities, mean (SD)	4.7 (2.3)	4.2 (2.5)	4.8 (2.1)
Hospital admission diagnosis (ICD-10), n (%)			
Mental and behavioral disorders	4 (4.5)	2 (4.5)	2 (4.5)
Diseases of the nervous system	5 (5.7)	2 (4.5)	3 (6.8)
Diseases of the eye and adnexa/ear and mastoid process	6 (6.8)	5 (11.4)	1 (2.3)
Diseases of the circulatory system	31 (35.2)	16 (36.4)	15 (34.1)
Diseases of the respiratory system	14 (15.9)	8 (18.2)	6 (13.6)
Diseases of the musculoskeletal system and connective tissue	5 (5.7)	4 (9.1)	1 (2.3)
Diseases of the genitourinary system	9 (10.2)	2 (4.5)	7 (15.9)
Other diseases	14 (15.9)	5 (11.4)	9 (20.5)
Fall since discharge, n (%)	25 (28.4)	11 (25.0)	14 (31.8)
Physical function:			
Short Physical Performance Battery, mean (SD)^a^	8.7 (2.3)	8.6 (2.3)	8.9 (2.2)
Gait speed m/s, mean (SD), *n = 85 (42 int. group and 43 cont. group)*	0.8 (0.2)	0.8 (0.2)	0.8 (0.2)
Grip strength kg, mean (SD)	26.3 (9.1)	26.8 (8.7)	25.8 (9.5)
Berg Balance Scale, mean (SD)^a^	48.9 (6.8)	48.8 (6.6)	48.9 (7.1)
6-min walk test m, mean (SD)	387.4 (115)	378.8 (109.8)	396.1 (120.7)
Body mass index, mean (SD)	26.9 (5.4)	26.1 (5.4)	27.8 (5.4)
International physical activity questionnaire (IPAQ), n = 69			
High, n (%)	7 (10.2)	4 (10.8)	3 (9.4)
Moderate, n (%)	25 (36.2)	14 (37.8)	11 (34.4)
Low, n (%)	37 (53.6)	19 (51.4)	18 (56.3)
Health related quality of life (SF-36):^b^			
Physical functioning, *n = 88 (44 int. group and 44 cont. group)*	58.9 (23.3)	57.1 (22.7)	60.7 (24.0)
Role physical, *n = 83 (41 int. group and 42 cont. group)*	47.0 (27.8)	42.9 (27.5)	51.0 (27.9)
Bodily pain, *n = 83 (42 int. group and 41 cont. group)*	56.6 (25.9)	55.8 (28.4)	57.4 (23.5)
General health, *n = 85 (43 int. group and 42 cont. group)*	52.2 (21.6)	50.5 (21.2)	54.1 (22.2)
Vitality, *n = 86 (43 int. group and 43 cont. group)*	44.9 (17.5)	44.8 (15.5)	44.9 (19.6)
Social functioning, *n = 87 (44 int. group and 43 cont. group)*	67.5 (28.1)	67.3 (26.9)	67.7 (29.5)
Role emotional, *n = 83 (43 int. group and 40 cont. group)*	59.4 (23.2)	62.2 (21.9)	56.5 (24.4)
Mental health, *n = 85 (43 int. group and 42 cont. group)*	67.7 (14.4)	66.7 (15.2)	68.7 (13.6)
Physical component summary, *n* = 79 *(40 int. group and 39 cont. group)*	39.0 (10.1)	37.3 (10.5)	40.7 (9.5)
Mental component summary, *n =* 79 *(40 int. group and 39 cont. group)*	47.7 (8.1)	48.0 (8.7)	47.3 (7.5)

*N* number of individuals, *ICD* International Classification of Disease, *BMI* Body Mass Index, calculated using the formula weight in kilograms divided by height in meters squared.

SF-36 = the medical Outcome 36 –Item Short Form Survey

^a^Higher scores reflect better physical function

^b^Higher scores reflect better HRQOL

Eighty-one patients (92%) had two or more comorbidities. Half of the participants walked less than 400 m on the 6-min walk test. Sixty percent scored ≤9 points on SPPB, scores ranged from 4 to 12. No floor- and ceiling effects occurred at baseline. At 4 months follow-up, 22.7% of the participants in the intervention group scored 12 on SPPB and 56 on BBS.

### ITT analyses

Table 2 presents the ITT analyses. No significant between group difference was found in the primary outcome: SPPB sum score (mean difference 0.8 points, 95% CI − 0.3-1.8, *p* = 0.151, effect size = 0.38). The results for the three subtests of SPPB can be seen in Additional file 6. There was a significant between group difference in favour of the intervention group on 6-min walk (mean difference 30.9 m, 95% CI 2.1–59.8 m, *p* = 0.036, effect size = 0.56) and the physical component summary of SF-36 (mean difference 7.1 points, 95% CI 3.1–11.1, *p* = 0.001, effect size = 0.94). No statistically significant differences between the groups were found in the mental component summary of SF-36, Berg Balance Scale, grip strength or BMI (Table 2). ITT analyses conducted on the dataset with imputations gave similar findings with respect to the between group differences (Additional file 4).

**Table 2:** Results at 4-month follow-up and effect of intervention based on intention-to-treat analysis

	Intervention group 4 months, mean (SD)	Control group 4 months, mean (SD)	Mean between group difference^a^	95% confidence interval	*P* value	Effect size^d^
Physical function						
SPPB^b^	9.3 (2.8)	9.3 (2.7)	0.8	−0.3 – 1.8	0.151	0.38
6-min walk test (m)	419.3 (122.9)	412.7 (138.3)	30.9	2.1 – 59.8	**0.036**	0.56
Berg Balance Scale^b^	50.0 (7.0)	50.5 (7.7)	−0.6	−2.2 – 0.9	0.402	0.22
Grip strength (kg)	28.0 (8.3)	26.5 (9.8)	1.1	−0.5 – 2.7	0.178	0.36
BMI	25.9 (3.5)	27.1 (5.9)	0.1	−0.4 – 0.6	0.672	0.12
HRQOL (SF-36)						
PCS^c^	42.9 (11.2)	38.5 (10.3)	7.1	3.1 – 11.1	**0.001**	0.94
MCS^c^	48.4 (8.1)	49.8 (7.8)	−0.7	−4.0 – 2.7	0.694	0.10

*SPPB* Short Physical Performance Battery, *BMI* Body Mass Index, calculated using the formula weight in kilograms divided by height in meters squared, *SD* Standard deviation, *HRQOL* Health Related Quality of Life, *SF-36* the medical Outcome 36 –Item Short Form Survey, *PCS* Physical component summary, *MCS* Mental component summary. Statistically significant *p*-values are in bold. The level of significance was set at 0.05

^a^Mean between group difference refers to difference between outcome at baseline and 4-month-follow up

^b^Higher scores reflect better physical function

^c^Higher scores reflect better HRQOL

^d^Effect size = Cohen’s d

## Discussion

Results from this study showed that a high intensity multicomponent exercise program did not improve physical performance measured by SPPB in older adults with or at risk of mobility disability after discharge from hospital. However, improvements in functional capacity measured by 6MWT and physical HRQOL were found. Further, this exercise program can be considered safe for this population since no serious adverse events occurred.

The lack of statistically significant difference in change between the groups in the primary outcome SPPB can be due to both the intervention group and the control group receiving instruction in home-based exercises and recommendations about physical activity for people aged 65+. However, this possible explanation does not match the significant difference in 6MWT. Another possible reason may be that SPPB is not as sensitive to change as the 6MWT [44]. Furthermore, the insufficient sample size may have caused a type 2 error, where a difference between the groups cannot be detected [45]. This also implies for all the secondary outcomes where no statistically significant difference in change between the groups was found. The lack of significant difference in SPPB and Bergs Balance Scale could possibly be attributed to a ceiling effect, since 22.7% of the participants in the intervention group achieved the highest possibly score after the intervention on both tests. Even though the between group difference in change in SPPB score was not statistically significant, a change of 0.8 points can be considered clinically meaningful [41].

In this study, the mean between group difference in change from baseline to four-month follow-up in distance walked in six minutes was 30.9 (2.1–59.8) meters. This is considered a small meaningful change in older people [41], and hence of clinical relevance. The amount of change in SF-36 that constitutes a meaningful change in older people has not been established, but a change of half a standard deviation may serve as a default value for important changes in HRQOL in different populations [46]. Hence, the change of 7.1 points in PCS in the present study can be considered clinically important.

The study sample had generally low scores on SPPB at baseline, compared to age-matched Norwegian reference values [44]. Further, the participants scored generally worse on HRQOL (SF-36) at baseline when compared to a normative sample of older people aged 70–80 [47]. This reinforce that older patients with or at risk of mobility disability while hospitalized have increased risk of transitioning to frailty, and possibly reduced independence and HRQOL. Targeting this group of older people in interventions aiming to improve physical function and HRQOL is, thus, of paramount importance in order to maintain their independence and HRQOL.

The present study extends the work done by Brovold et al., a randomised controlled trail comparing an aerobe high intensity exercise program with usual care in a Norwegian sample of 115 independent older people recently discharged from hospital [4]. Our results are in line with this study that also found a significant between group difference in 6 min walk test. However, whereas Brovold et al. excluded participants who could not complete the Timed up and go test within 20 s, we included mobility limited participants with a score ≤ 9 points on the SPPB. Older adults with or at risk of mobility disability after discharge from hospital is a group of people that is often excluded from studies, despite being at high risk for many negative outcomes.

This study adds to previous research suggesting that group-based exercises can be beneficial and safe to older people after discharge from hospital [15, 20]. This study brings new knowledge about the effect of an exercise program on functional capacity and physical HRQOL to the population of older adults with or at risk of mobility disability after discharge from hospital. According to McKelvie et al., such studies are urgently needed [18].

The most important limitation of this study is the low number of participants. We experienced that it was difficult to recruit participants to the study, a large portion of the eligible patients declined to participate, and we did not accomplish the estimated sample size. As already mentioned, the lack of power might have prevented significant findings regarding between group changes. According to Buurman [48] the problem of declining participants is frequently encountered in studies recruiting acutely hospitalized older people. The inclusion rate might have implications for the generalizability of the study results, but we found no significant differences in age and sex between those who declined participation and those who accepted inclusion.

The difficulties of recruiting patients could be an indication that a major part of the targeted group should be offered another alternative when discharged from hospital. In accordance with the most common reasons for declining to participate, offering a more flexible program with drop-in classes could be an alternative. Another option could be to offer a supervised home-based exercise program to the patients who declined to participate due to traveling time/logistics. However, the effect of a supervised high intensity multicomponent home-based exercise program aiming to increase physical function and HRQOL has to the best of our knowledge not been studied in this population and should be tested in a feasibility study and further in a randomized controlled trial.

The participants who were included accepted to participate in a multicomponent high-intensity exercise trial aiming to increase their physical function and HRQOL. This may have caused selection-bias of the most fit and motivated patients [49], and limit the generalisation of the results. Further, the participants were recruited from only one hospital in Oslo, and the study sample may not be representative for the general population of older people in Norway.

Finally, interventions that start while the older adult is inpatient and continue after discharge is recommended for this population [50]. The short length of stay in Norway makes that challenging to accomplish, but further studies should try to start as early as possible after admission.

## Conclusion

In conclusion, the high-intensity multicomponent exercise program significantly improved functional capacity and physical HRQOL in this sample of older people with or at risk of mobility disability after discharge from hospital.

## Supplementary information

**Additional file 1.** The high intensity multicomponent exercise program described according to the Consensus on Exercise Reporting Template (CERT) guidelines.

**Additional file 2.** Information sheet: recommendations on physical activity for people 65 years and above

**Additional file 3.** Home exercises.

**Additional file 4.** Results at 4-month follow-up and effect of intervention based on intention-to-treat analysis with multiple imputations.

**Additional file 5.** CONSORT 2020 Checklist

**Additional file 6.** The subcomponents of SPPB

## Data Availability

The datasets used and/or analysed during the current study are available from the corresponding author on reasonable request.

## Abbreviations

HRQOL: Health-Related Quality of LIFE
SPPB: Short Physical Performance Battery
6MWT: 6-min walk test
BBS: The Berg Balance Scale
BMI: Body Mass Index
SF-36: The Medical Outcome Study 36 Item Short-Form Health Survey
WHO: World Health Organization
RM: Repetition Maximum
NTNU: Norwegian University of Science and Technology
IPAQ-SF: International Physical Activity Questionnaire – Short form
ITT: Intention-To-Treat
PF: Physical function
SD: Standard Deviations
N: Numbers
ICD: International Classification of Disease
