# Supplementary material for: Effects of a multicomponent high intensity exercise program on physical function and health-related quality of life in older adults with or at risk of mobility disability after discharge from hospital: a randomised controlled trial
Source: BMC Geriatr. 2020 Nov 11;20:464. doi: 10.1186/s12877-020-01829-9 (PMC7656746; doi:10.1186/s12877-020-01829-9)
Supplement: Supplementary file 1 — Additional file 1. The high intensity multicomponent exercise program described according to the Consensus on Exercise Reporting Template (CERT) guidelines. [file 12877_2020_1829_MOESM1_ESM.docx]

**Additional file 1.** The high intensity multicomponent exercise program described according to the Consensus on Exercise Reporting Template (CERT) guidelines. *Slade SC, Dionne CE, Underwood M, et al, Consensus on Exercise Reporting Template (CERT): Explanation and Elaboration Statement. British Journal of Sports Medicine 2016;****50:****1428-1437.*

| Item # | Description | Page |
| --- | --- | --- |
| 1 | Parallel bar, chairs, weight belts loaded with op to 12 kg, Reebok® Steps, one soft ball, Spotify playlist. | 6 |
| 2 | Seven physiotherapists delivered the intervention, with clinical experience ranging from one to 28 years. All physiotherapists scrutinized the detailed intervention manual, and participated at least five intervention sessions together with the project leader (S.S.) | 6 |
| 3 | The participants exercised in groups of up to ten people. The groups were running continuously, with participants in different stages of their 16 weeks period. All participants were subscribed 32 sessions, twice a week for 16 weeks. To compensate for possible absence, we allowed attendance for maximum 20 weeks to attend up to 32 sessions. | 6 |
| 4 | All exercise sessions were supervised and the whole session was instructed by a physiotherapist. When one or more participants required support due to low physical function or mental capabilities, a second physiotherapist or physiotherapy student co-instructed the session to ensure safety. A second physiotherapist also attended sessions where more than one person commenced at the same time, to ensure individual tailoring and instructions for those who were new to the intervention. | 6 |
| 5 | Adherence to exercise was recorded in an attendance scheme at every session by the physiotherapist who instructed the group. Those participants who attended at least 16 sessions were defined as adherent to the intervention.  Adherence to the home exercises was measured by asking the participants to fill out an exercise diary. The first 20 participants who performed testing at four months follow-up completed the diary. Several of the participants reported dissatisfaction with the reporting (too time-consuming, not feeling relevant etc.). In addition, we found that the details needed to report adherence were not sufficiently described by quite a few participants. Hence, we decided to omit the exercise diary for the rest of the participants. | 10 |
| 6 | The physiotherapist (-s) motivated the participants verbally during the exercise sessions, and physically by participating in the program and exercise at high intensity during the endurance and strength exercises. Phone calls were made to those who did not show up for the intervention for unknown reasons more than two subsequent sessions, to motivate for returning to the group. Participants were encouraged to attend the sessions and take part in as much of the program as they could tolerate, if they did not feel quite well. They were encouraged to take breaks whenever they needed, and chairs were available for rest. | 6 |
| 7a | The duration of the intervention is 16 weeks and not the usual 12 weeks because experience from a previous study suggests that older people need longer follow-up time to gradually adjust to exercise intensity. The participants were told to exercise with a low to moderate intensity the first four sessions, to minimize the risk of muscle soreness and other adverse events. They wore the weight belts without any weights. They were then encouraged to take a gradual approach to increase intensity to a moderate to high level (15-18 out of 20 on Borg rating scale of perceived exertion (1), which corresponds to up to approximately 90 % maximum heart rate). For the strength exercises the goal was to perform two sets of 8-12 RM and the exercise intensity was individualized. The participants had their designated weight belt, and were encouraged to increase the load as soon as they could conduct 8-12 RM. The weight belts were stored with the chosen amount of weights for the next session. For the balance exercises, the participants were encouraged to exercise near the limits of maintaining postural stability. | 6 |
| 7b | The exercise program was progressed based on the participants perceived intensity level and observation of the physiotherapist. The physiotherapist repeated information about the necessity of progress to improve every session and encouraged the participants to push their limits on days they felt in good health/that their health condition allowed it. Progression was made by increasing the range of motion, including arm movements, weights (load in the weight belts), smaller base of support in balance, or more challenging throwing of the ball. | 6 |
| 8 | \| Exercises \| Therapeutic goal \| Description \| Level \| Progression or modify \| \| --- \| --- \| --- \| --- \| --- \| \| Exercises during warm-up period Song 1-2 \| Warm-up and flexibility exercises \| Marching on the spot  Exercises using arm movements and including breathing (inhaling while raising arms, exhaling while lowering arms)  Walking one step sideways to the right and back  Lateral trunk stretches  Stationary walking: alternate heel touches the floor in front of the body  Shoulder circle exercises  Squats \| 1  2  3 \| Hold on to a steady subject for support (parallel bar, wall, chair)  Including arm movements  Increase ROM \| \| Exercises during high-intensity periods (songs 3-4, 7-8 and 12-13) \| Improve functional capacity and balance \| Walking in a circle  Walking 4 steps forwards and 4 steps backwards  Walking 2 steps sideways to the left, and 2 steps sideways to the right  Marching on the spot  Knee lifts  Knee flexion behind the body (heel towards glut. max) \| 1  2  3 \| Hold on to a steady subject for support (parallel bar, wall, chair)  Including arm movements  Increase ROM \| \| Exercises during flexibility and balance periods (song 5, the beginning of song 6, song 9-10 and 14) \| Improve flexibility and balance.  Cool down period \| Arm circle exercises  Exercises using arm movements and including breathing (inhaling while raising arms, exhaling while lowering arms)  Walk over a step forward and sideways  Catching and throwing a ball  Seated trunk rotation. Exercise number 7 in Home exercise program, (Additional file 3)  Swing and flex “skiing”  Shoulder circle exercises  Lateral lounges  Lateral trunk stretches  “Harvesting apples” Exercise number 5 in Home exercise program (Additional file 3)  Walk on a line. Exercise number 6 in Home exercise program  (Additional file 3)  One leg stand. Exercise number 3 in Home exercise program (Additional file 3)  Toe rises. Exercise number 2 in Home exercise program (Additional file 3) \| 1  2 \| Reduce base of support  Increase ROM \| \| Exercises during strength periods (song 6 and 11) \| Improve leg strength and balance \| Sit to stand (raise from a chair). Exercise number 1 in Home exercise program (Additional file 3)  Forward lounges. Exercise number 4 in Home exercise program (Additional file 3) \| 1  2  3 \| Hold on to a steady subject for support (parallel bar, wall, chair)  Increase load in weight belt  Increase ROM \| | 6 |
| 9 | Both groups received a home exercise program = Additional file 3. | 7 |
| 10 | Both groups received: Information sheet: recommendations on physical activity for people 65 years and above = Additional file 2. | 7 |
| 11 | The physiotherapists leading the intervention reported three falls without injury. One participant was confused (delirium) when showing up to a session and was admitted to the hospital. | 10 |
| 12 | The intervention was delivered in the hospital gym. Five participants received some sessions in a rehabilitation site in the municipality. | 5-6 |
| 13 | The exercise program started with an eight-minute warm-up period (song 1- 2) focusing on dynamic exercises of muscle groups in the upper- and lower extremities and gentle flexibility and breathing exercises. The warm-up progressed gradually to the first high intensity exercise interval, which constituted 9 minutes of walking exercises and stationary exercises (song 3- 4). Followed by an 8 minutes interval of moderate intensity (song 5 -6) containing flexibility, balance and strength exercises. Songs 7-8 constituted the next bout of high intensity exercises (7 minutes), followed by the second interval of moderate intensity (10 minutes) containing flexibility, balance and strength exercises (song 9-11). Song 12-13 made the last bout of high intensity exercise interval (6 minutes). Finally, song 14 (5 minutes) constituted cool down and flexibility exercises. The whole session lasted one hour, including 53 minutes of work interspersed with 3 x 2 minutes brakes for rest and drinking water after the high intensity exercise periods. | 6 |
| 14a | The high intensity multicomponent intervention program was designed based on the Norwegian Ulevaall model (2) and the Swedish High-Intensity Functional Exercise Program (the HIFE program) (3, 4). In addition to recommendations for exercise in older people in general (5), and for improving physical function in community-dwelling older adults with impaired mobility, physical disability and/or multi-morbidity (6). The exercises were tailored to match each participants level of physical function. | 6 |
| 14b | The exercises were tailored to each participant by offering a support object if the participant was using a walking aid or otherwise had difficulties with balance. The exercises were adapted to those who could not performed the exercises as subscribed. The physiotherapist encouraged the participants to progress when the level was below 15-18 on Borgs rating scale of perceived exertion for the high interval bouts, and to increase load in the weight belt when a participant could perform two sets of 8-12 repetitions with right technique without fatigue on the strength exercises. For the balance exercises the participants were encouraged to challenge their balance as much as possible without jeopardizing safety. The participants were standing close to a wall/chair or in a parallel bar to minimize the risk of falling. | 6 |
| 15 | The starting level of each participant was based on the results from the physical tests at baseline. Regardless of physical function, all participants were told to start with moderate intensity (Borg 11-13) the first two weeks. And then gradually and slowly increase intensity based on their experiences of any adverse events and perceived level of exertion. All exercises could be performed at different levels of difficulty. | 6 |
| 16a | Adherence was measured by registering attendance at each session, and by dividing the number of sessions a participant attended by 32 (subscribed number of sessions). Adherence regarding actual intensity accomplished was not measured. | 10 |
| 16b | The intervention was delivered as intended. The physiotherapist who designed the intervention (SS) developed a detailed description of the content of the intervention. All physiotherapists who held the intervention participated in the intervention together with the project leader at least five times before leading the intervention on their own. | 6 |

**References**

1. Borg G. Perceived exertion as an indicator of somatic stress. Scandinavian journal of rehabilitation medicine. 1970.

2. Nilsson BB, Lunde P, Holm I. Implementation and evaluation of the Norwegian Ullevaal model as a cardiac rehabilitation model in primary care. Disability and Rehabilitation. 2017;41(4):1-8.

3. Littbrand H, Rosendahl E, Lindelof N, Lundin-Olsson L, Gustafson Y, Nyberg L. A high-intensity functional weight-bearing exercise program for older people dependent in activities of daily living and living in residential care facilities: evaluation of the applicability with focus on cognitive function. Phys Ther. 2006;86(4):489-98.

4. Littbrand H, Lindelöf N, Rosendahl E. The HIFE program: the high-intensity functional exercise program. Umea: Universität Umea. 2014.

5. Garber CE, Blissmer B, Deschenes MR, Franklin BA, Lamonte MJ, Lee IM, et al. American College of Sports Medicine position stand. Quantity and quality of exercise for developing and maintaining cardiorespiratory, musculoskeletal, and neuromotor fitness in apparently healthy adults: guidance for prescribing exercise. Med Sci Sports Exerc. 2011;43(7):1334-59.

6. de Vries NM, van Ravensberg CD, Hobbelen JSM, Olde Rikkert MGM, Staal JB, Nijhuis-van Der Sanden MWG. Effects of physical exercise therapy on mobility, physical functioning, physical activity and quality of life in community-dwelling older adults with impaired mobility, physical disability and/or multi-morbidity: a meta-analysis. Ageing research reviews. 2012;11(1):136.
