# Supplementary material for: Effects of a multicomponent high intensity exercise program on physical function and health-related quality of life in older adults with or at risk of mobility disability after discharge from hospital: a randomised controlled trial
Source: BMC Geriatr. 2020 Nov 11;20:464. doi: 10.1186/s12877-020-01829-9 (PMC7656746; doi:10.1186/s12877-020-01829-9)

**Additional file 2.** Information sheet: recommendations on physical activity for people 65 years and above, URL: <https://helsenorge.no/SiteCollectionDocuments/Nasjonale%20anbefalinger%2065%20pluss.pdf>

Accessed June 12, 2020


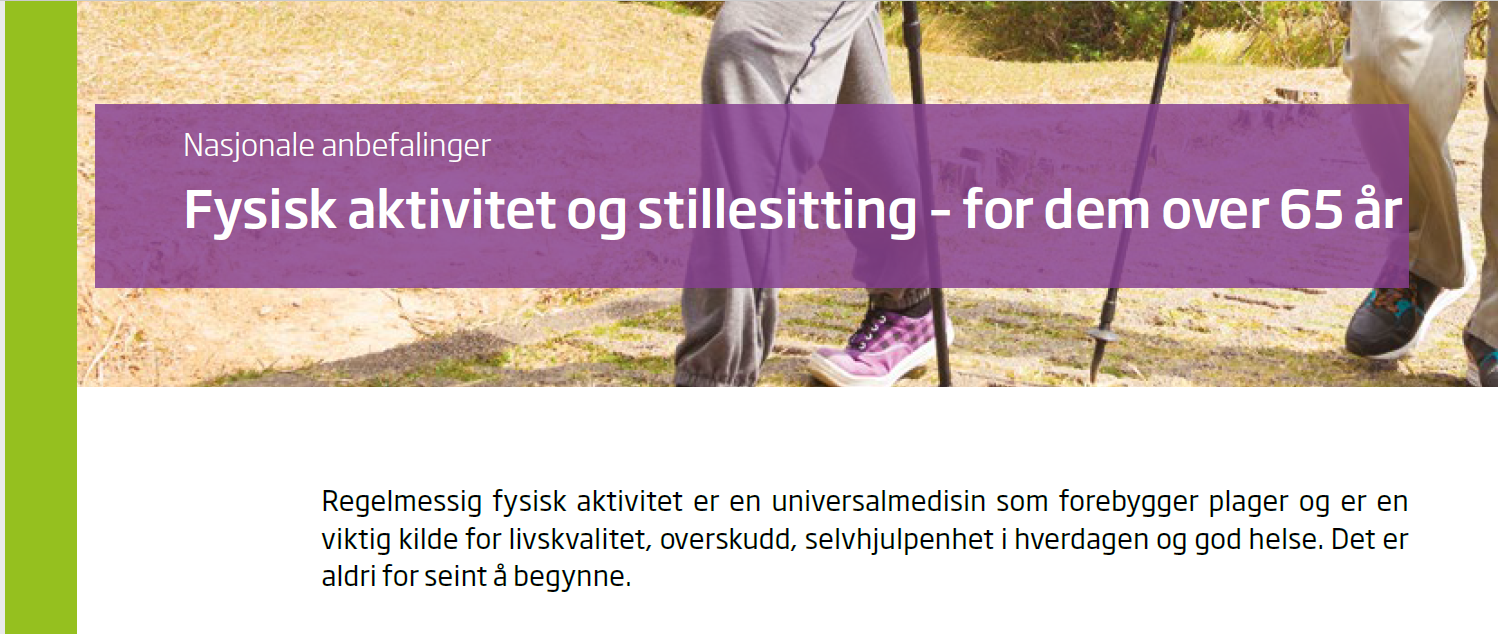


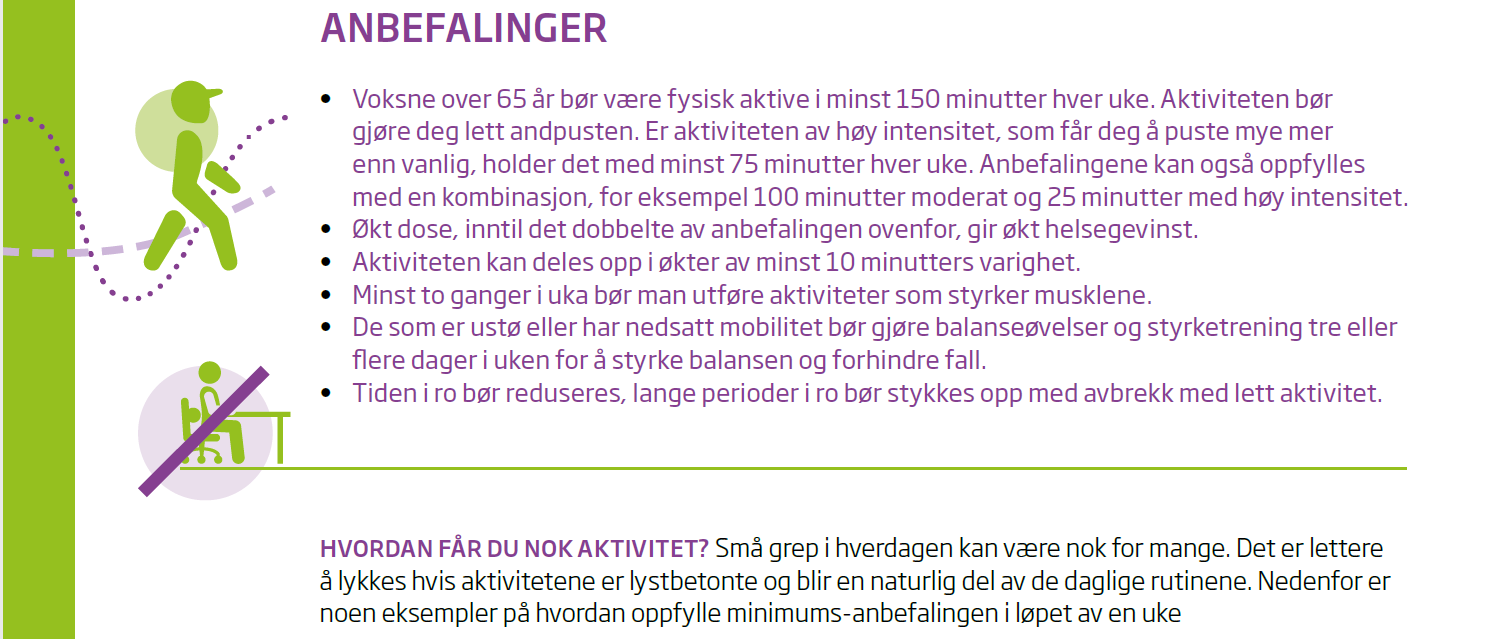


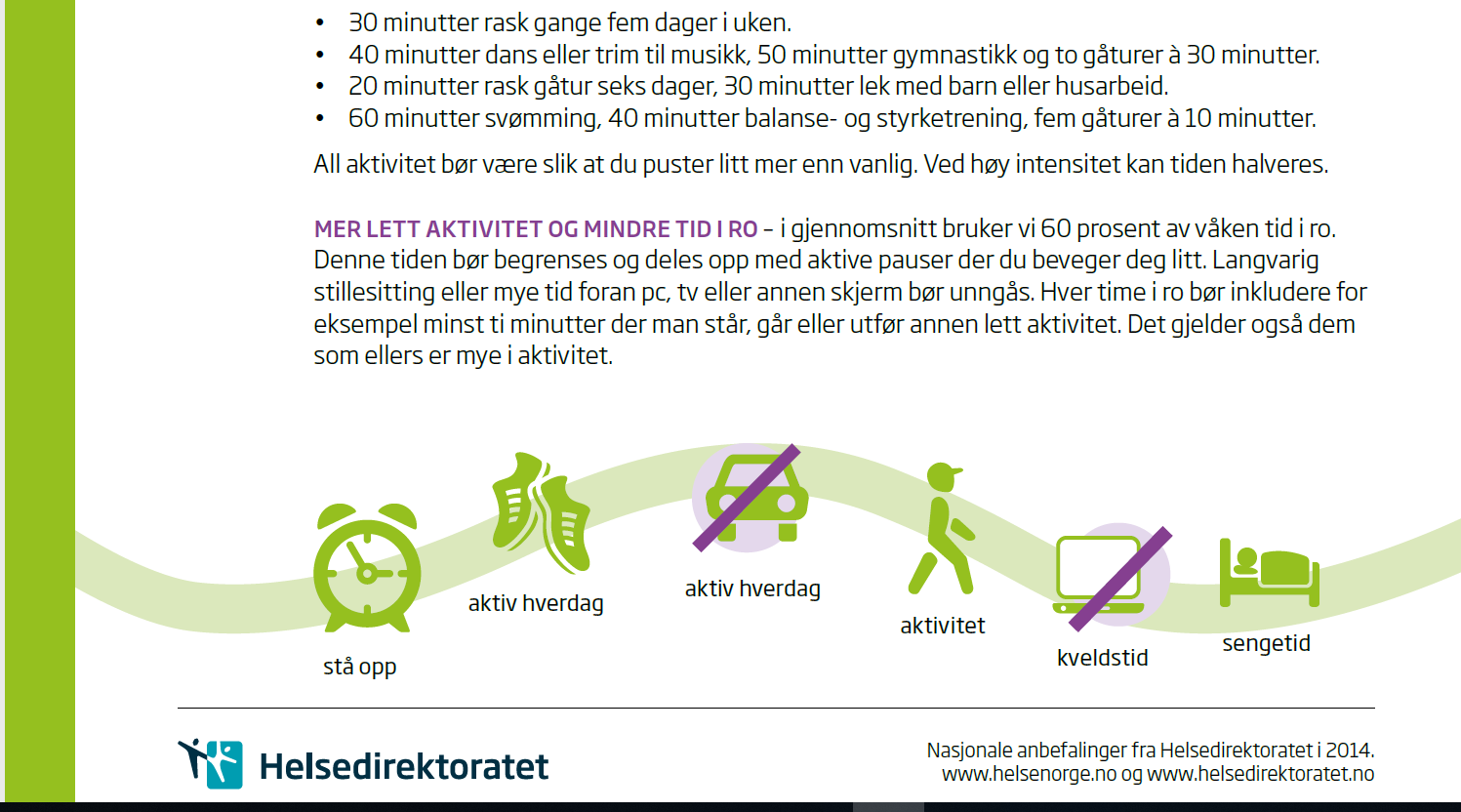

Supplement: Supplementary file 2 — Additional file 2. Information sheet: recommendations on physical activity for people 65 years and above [file 12877_2020_1829_MOESM2_ESM.docx]
