# Supplementary material for: Effects of a multicomponent high intensity exercise program on physical function and health-related quality of life in older adults with or at risk of mobility disability after discharge from hospital: a randomised controlled trial
Source: BMC Geriatr. 2020 Nov 11;20:464. doi: 10.1186/s12877-020-01829-9 (PMC7656746; doi:10.1186/s12877-020-01829-9)

**Additional file 3.** Home exercises, URL: <https://helsenorge.no/SiteCollectionDocuments/ntnu_fallforebygging_1_2016-ny2.pdf>

Accessed 8 June 2020


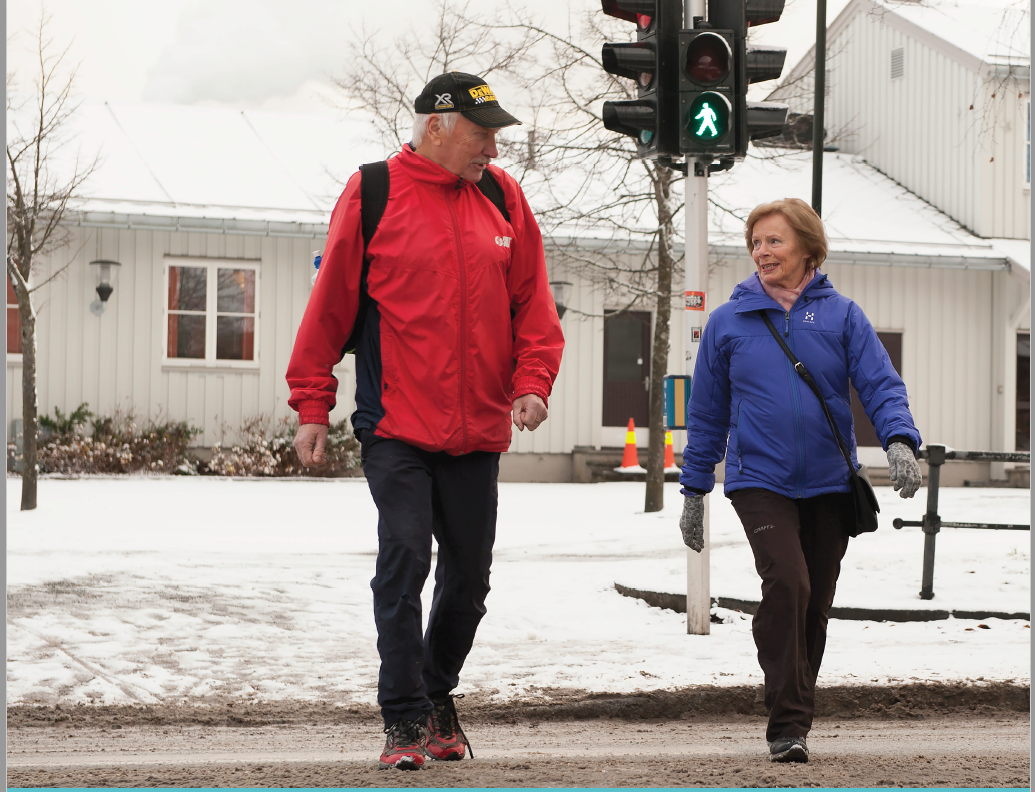


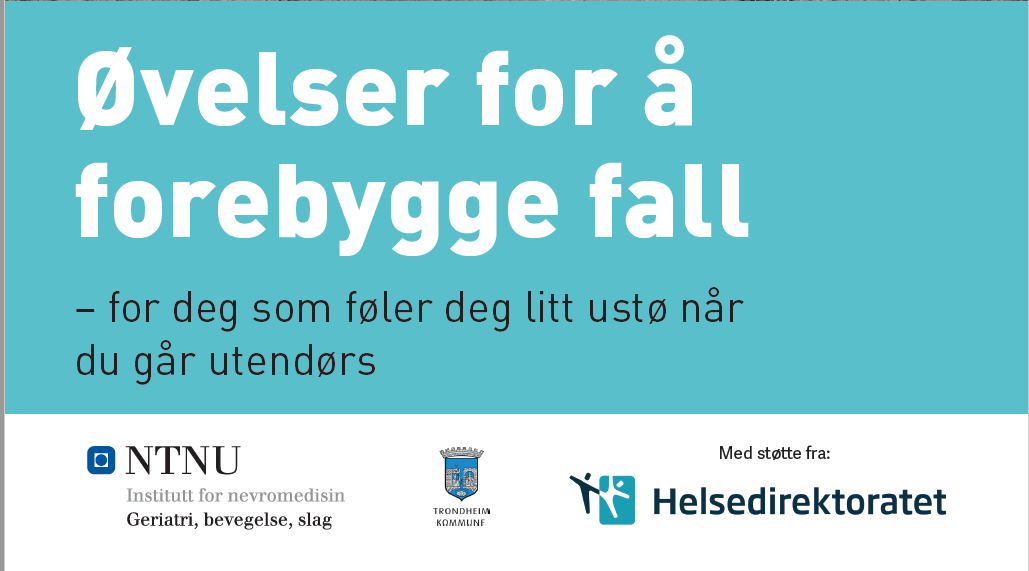


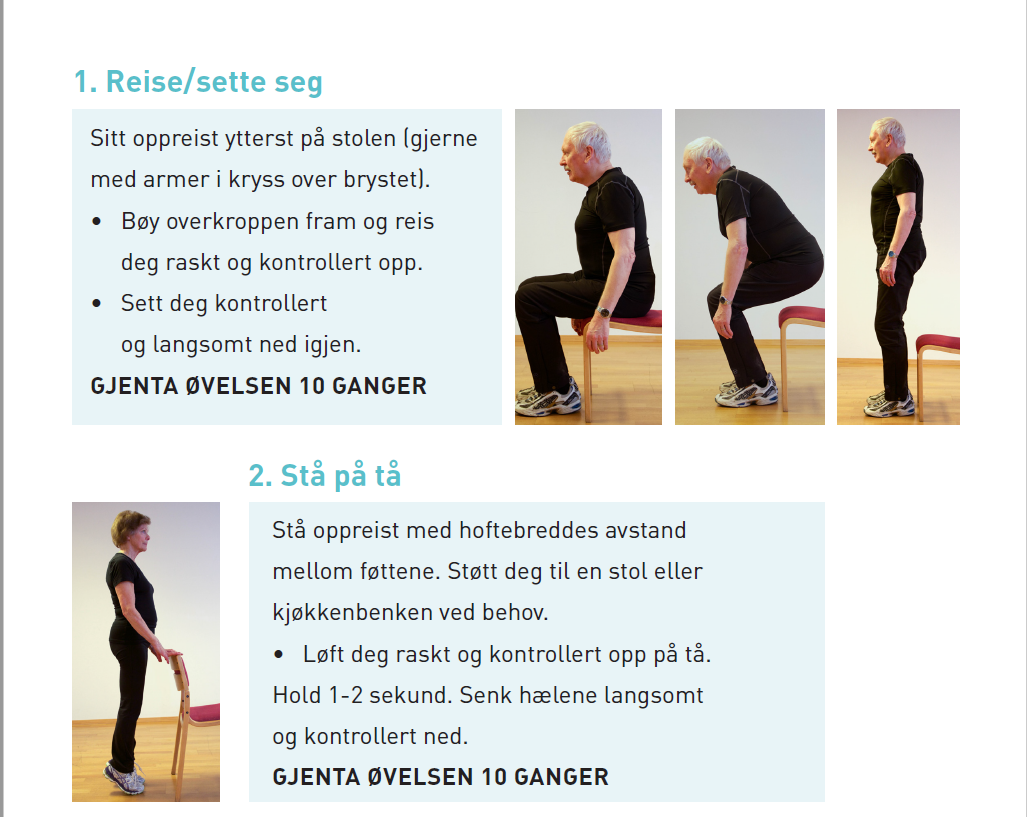


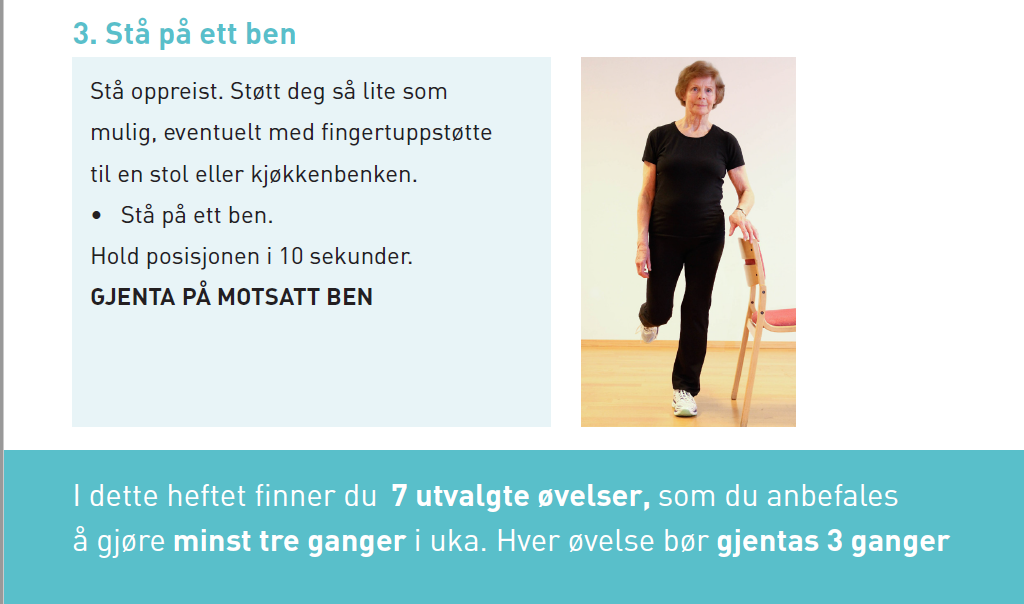


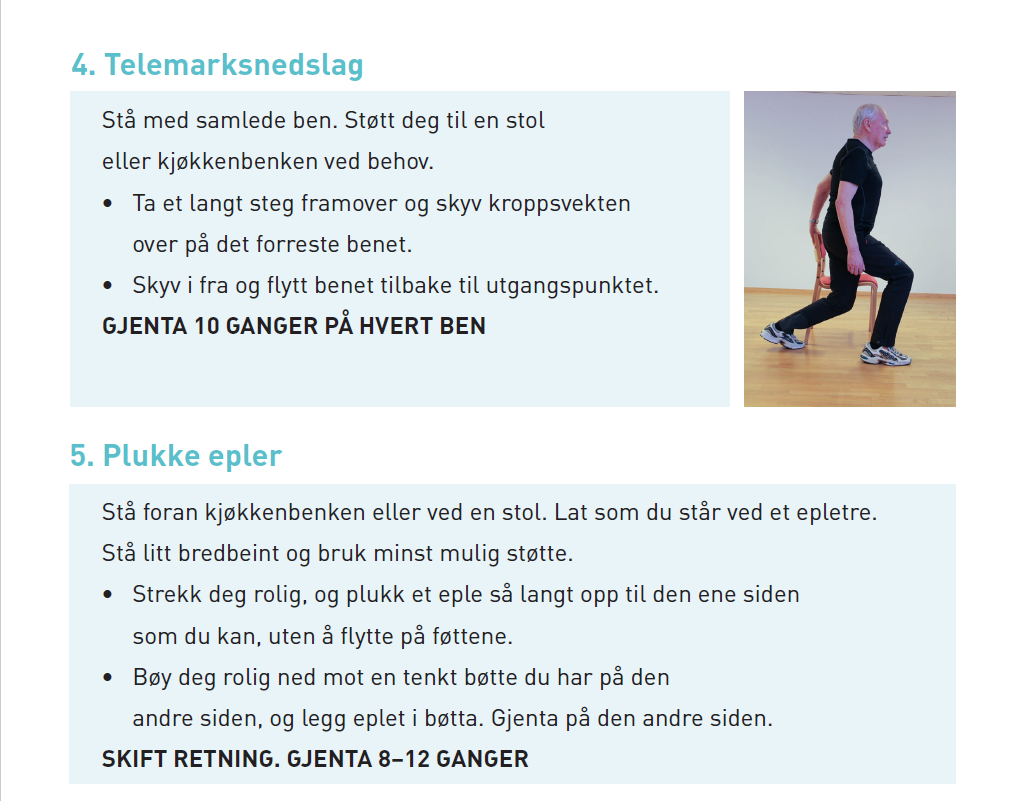


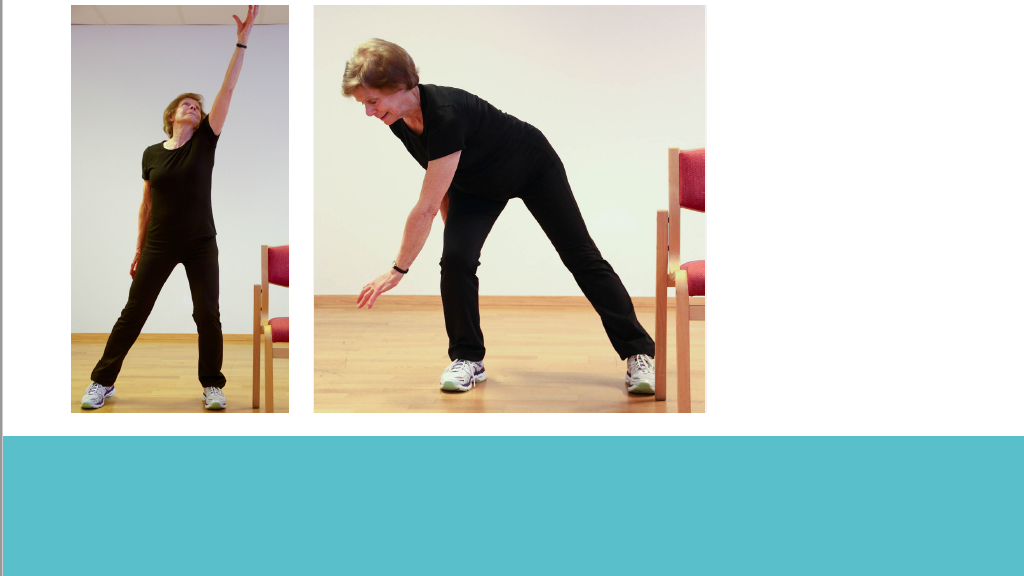


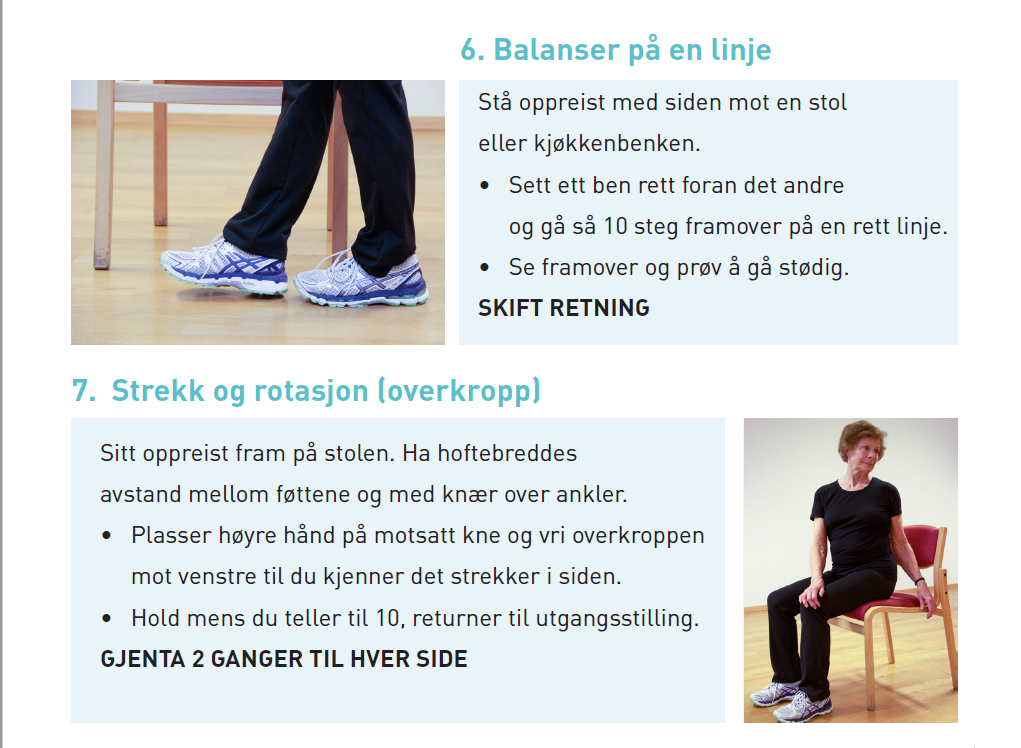


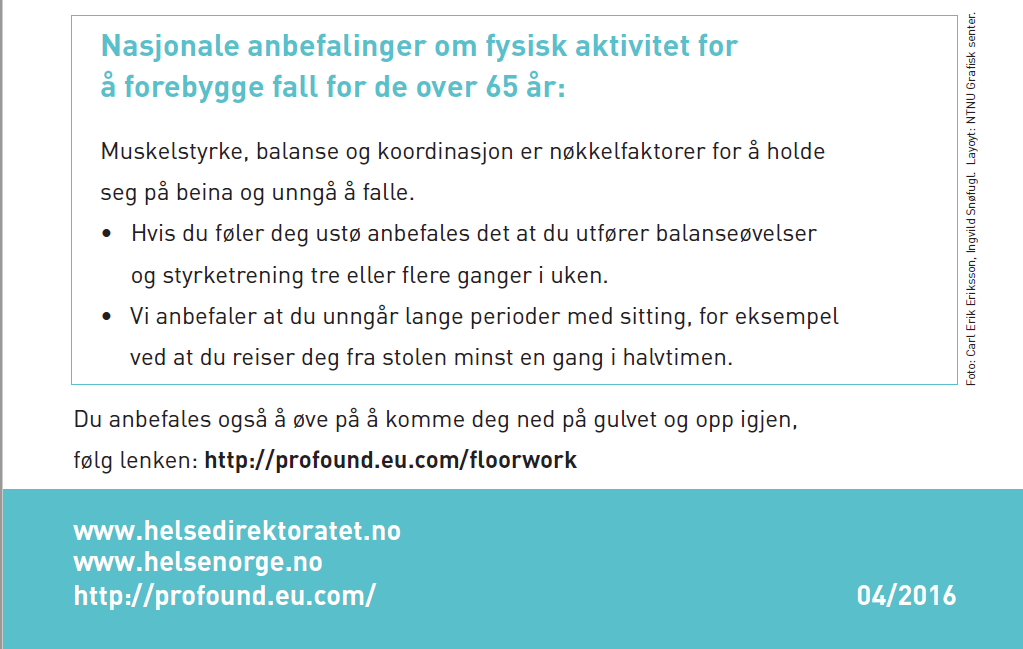

Supplement: Supplementary file 3 — Additional file 3. Home exercises. [file 12877_2020_1829_MOESM3_ESM.docx]
