# Supplementary material for: Effects of a multicomponent high intensity exercise program on physical function and health-related quality of life in older adults with or at risk of mobility disability after discharge from hospital: a randomised controlled trial
Source: BMC Geriatr. 2020 Nov 11;20:464. doi: 10.1186/s12877-020-01829-9 (PMC7656746; doi:10.1186/s12877-020-01829-9)
Supplement: Supplementary file 4 — Additional file 4. Results at 4-month follow-up and effect of intervention based on intention-to-treat analysis with multiple imputations. [file 12877_2020_1829_MOESM4_ESM.docx]

**Additional file 4.** Results at 4-month follow-up and effect of intervention based on intention-to-treat analysis with multiple imputations.

|  | Intervention group 4 months mean (SD) | Control group 4 months mean (SD) | Mean difference | 95% confidence interval | *P* value |
| --- | --- | --- | --- | --- | --- |
| Physical function |  |  |  |  |  |
| SPPB | 9.3 (2.8)^b^ | 9.3 (2.7) | 0.6 | -0.2 – 1.5 | 0.137 |
| 6-minute walk test | 419.3 (122.9) | 412.7 (138.3) | 20.2 | 1.2 – 39.3 | **0.037** |
| Berg Balance Scale | 50.0 (7.0) | 50.5 (7.7) | -0.4 | -1.5 – 0.7 | 0.466 |
| Grip strength kg | 28.0 (8.3) | 26.5 (9.8) | 0.6 | -0.6 – 1.7 | 0.340 |
| BMI | 25.9 (3.5) | 27.1 (5.9) | 0.1 | -0.3 – 0.6 | 0.637 |
| HRQOL (SF-36) |  |  |  |  |  |
| PCS | 42.9 (11.2) | 38.5 (10.3) | 4.5 | 1.5 – 7.5 | **0.004** |
| MCS | 48.4 (8.1) | 49.8 (7.8) | -0.4 | -2.7 – 1.9 | 0.729 |

SPPB = Short Physical Performance Battery. BMI = Body Mass Index, calculated using the formula weight in kilograms divided by height in meters squared. SD =Standard deviation. HRQOL = Health Related Quality of Life. SF-36 = the medical Outcome 36 –Item Short form Survey. PCS = Physical component summary. MCS = Mental component summary. Statistically significant p-values are in bold. The level of significance was set at 0.05. ^a^Mean difference refers to difference between outcome at baseline and 4-month-follow up.
