# Supplementary material for: Effects of a multicomponent high intensity exercise program on physical function and health-related quality of life in older adults with or at risk of mobility disability after discharge from hospital: a randomised controlled trial
Source: BMC Geriatr. 2020 Nov 11;20:464. doi: 10.1186/s12877-020-01829-9 (PMC7656746; doi:10.1186/s12877-020-01829-9)
Supplement: Supplementary file 6 — Additional file 6. The subcomponents of SPPB [file 12877_2020_1829_MOESM6_ESM.docx]

Table 2. Results at 4-month follow-up and effect of intervention based on intention-to-treat analysis.

|  | Intervention group 4 months,  mean (SD) | Control group 4 months,  mean (SD) | Mean between group difference^a^ | 95% confidence interval | *P* value | Cohens d |
| --- | --- | --- | --- | --- | --- | --- |
| SPPB |  |  |  |  |  |  |
| Balance^b^ | 3.14 (1.2) | 3.43 (1.0) | 0.17 | -0.32 – 0.66 | 0.491 | 0.18 |
| 4 meter walk^b^ | 3.5 (0.7) | 3.4 (1.0) | -0.34 | -0.71 – 0.03 | 0.067 | 0.49 |
| Sit to stand^b^ | 2.7 (1.5) | 2.4 (1.3) | -0.58 | -1.22 – 0.06 | 0.074 | 0.47 |

SPPB = Short Physical Performance Battery. Statistically significant p-values are in bold. The level of significance was set at 0.05. ^a^Mean between group difference refers to difference between outcome at baseline and 4-month-follow up.

^b^ Higher score reflect better physical function.
